# Adapting a selective parent-focused child sexual abuse prevention curriculum for a universal audience: A pilot study

**DOI:** 10.1371/journal.pone.0302982

**Published:** 2024-05-16

**Authors:** Kate Guastaferro, Vanessa Abuchaibe, Kaylee V. McCormick, Arushee Bhoja, Ella Abourjaily, Mia Melchior, Corinne Grayson, Paige Welikson, Colin Dan, Meron B. Zeleke

**Affiliations:** 1 School of Global Public Health, New York University, New York, New York, United States of America; 2 Mission Kids Child Advocacy Center, East Norriton, Pennsylvania, United States of America; The University of Alabama, UNITED STATES

## Abstract

Parents are an obvious, but underutilized player in the prevention of child sexual abuse (CSA). A handful of universal parent-focused prevention programs have emerged, however, the evidence for these programs is mixed and the programs suffer ubiquitously from barriers to implementation (e.g., poor engagement, low participation) thereby limiting public health impact. To combat these barriers and improve evidence, researchers previously developed and tested a selective parent-focused CSA prevention program. While promising, the selective approach still leaves a gap in the prevention landscape–parents from the universal audience. However, there appear to be no standardized methods to inform this type of adaptation—interventions designed as universal or selective have primarily been delivered as such. This study sought to adapt the selective curriculum for a universal audience and examined the acceptability and feasibility of the program for evaluation in a future trial. Using mixed methods, N = 31 parents (i.e., primary caregiver for a child under 13) completed pre- and post-workshop surveys followed by a brief individual interview conducted via Zoom. Interviews, coded using content analysis methods, focused on three themes: parents as agents of prevention (e.g., prior action, confidence), curriculum (e.g., content, design), and engagement (e.g., future marketing and promotion). Overall participants’ mean score on CSA-related awareness and intention to use protective behavioral strategies increased. The participants found the curriculum highly acceptable noting strengths in the content and design. All told, the results of this pilot study suggest the acceptability and feasibility of examining the efficacy of the universal parent-focused curriculum in a larger trial. Procedural challenges, such as bots in recruitment, identify areas of caution in design of the larger trial and a roadmap for others seeking to adapt selective programs for universal audiences.

## Introduction

Child sexual abuse (CSA) is a public health concern of considerable magnitude. National prevalence estimates report over 60,000 confirmed cases of CSA every year in the U.S., though the true prevalence is believed to be much higher [1,2]. The experience of CSA is linked to myriad adverse lifelong psychological [3,4], behavioral [5–7], and physical health outcomes [8–10] and confers a lifetime economic burden estimated to exceed $9.3 billion USD [11]. Thus, prevention of CSA is a public health priority. Primary prevention programs are those that limit exposure to risk for maltreatment, secondary programs are designed to address identified risk factors before maltreatment occurs, and tertiary programs are designed to mitigate the consequences of maltreatment that has occurred and to prevent repeated instances of maltreatment [12]. These points of intervention require specific strategies. Typically, as noted by Jones-Harden et al. [13], primary prevention efforts adopt a *universal* approach (i.e., geared to the whole population with no evidence of maltreatment) whereas secondary efforts use *selective* strategies (i.e., targeting individuals with elevated risk) and tertiary efforts use *indicated* preventive interventions (i.e., aimed at reducing recurrence).

The most pervasive primary prevention efforts are universal-school based programs targeting elementary school-age children with the philosophy of ‘hardening the target.’ These school-based interventions prioritize increasing children’s CSA knowledge and protective behaviors [12]. In recent years, there has been a call not to place the ‘burden of prevention’ on children alone. Several community-based, adult-focused, interventions have emerged [13–17]. These community-based primary prevention efforts often include raising awareness of CSA, challenging social norms, and increasing the ability to recognize signs of CSA [15]. Generally speaking, existing adult-focused prevention efforts are secondary–meaning reactive–in nature: CSA perhaps has occurred and the goal is to mitigate outcomes, or risk for CSA is high and efforts are enacted to reduce the potential risk. Despite their proliferation, child-focused and community-based prevention methods have failed to empirically demonstrate notable effects on prevalence rates of CSA. While children and community members play an important role in the prevention of CSA, there remains a pressing need for a comprehensive, multilevel approach to this public health issue. There is an obvious player missing from current CSA prevention efforts: parents.

Parents have also been successfully integrated in the prevention of other threats for the well-being of youth, including substance use [18], delinquency [19], and obesity [20,21]. However, specifically related to the prevention of CSA, parents (defined generally to include any adult in a primary caregiver role for children under 18; e.g., biological parent, step-parent, foster parent, aunt, uncle, etc.) have a unique role in creating and fostering protective environments for their children [22,23]. Parents have the ability to monitor activities and who has access to the child, countless opportunities to promote open and honest communication surrounding sexual topics from a young age, and the ability to encourage their child’s sense of competency and well-being, a protective factor from sexual abuse [22,24,25]. Parents may also indirectly prevent victimization through encouraging their child’s self-efficacy, self-esteem and confidence [25]. Given the prevalence and magnitude of CSA, there is an obvious, and urgent, public health need for primary (i.e., proactive) CSA prevention efforts targeting awareness and behaviors designed for universal parent audiences.

### Parent-focused CSA prevention efforts

A handful of universal parent-focused CSA programs exist; however, empirical studies of these programs are few and results are mixed. Preliminary research provides support for parental involvement in personal safety education. Several early studies demonstrated the majority of parents strongly support the education of children on CSA and parents are receptive to learning more about CSA [25–29]. A study that examined parents’ attitudes found that though most parents (64%) engaged in discussions about CSA, they still desired more information from trusted sources and needed developmentally appropriate materials [30]. Additionally, prior research has demonstrated that though parents generally are aware of CSA risks, they do not apply the recommended comprehensive prevention messages–instead, they are more likely to focus on the danger of kidnapping [31].

Translating knowledge and awareness into action has been difficult to demonstrate among the existing parent-focused prevention programs. A meta-analysis of 24 studies conducted by Rudolph and colleagues, investigated 18 parent-focused CSA prevention programs and suggested that results are mixed largely due to the product of the study design, as many are not designed to demonstrate efficacy (i.e., qualitative or post-test only) [23]. For example, in their review, only 58% (n = 14) assessed outcomes at pre- and post-intervention and only 17% (n = 4) included a follow-up (ranging from 1 month to 2-months). Related, evaluations of parent-focused CSA prevention programs have historically reported very low engagement (i.e., recruitment and retention). In the author’s review, attrition rate in existing CSA-prevention studies varied from 0% to 63%. These challenges may be attributed to some degree to the structure of the program including duration and number of sessions [23].

Programming of existing programs vary in structure (i.e., duration and format). For example, the *Stop It Now*! intervention entails watching a brief (less than 1 minute) informational video [15], the *Families Matter Program* includes a total of 18 hours of intervention over 6 weeks [32], and one program conducted in Turkey is delivered as a 1-day workshop delivered in a series of four stages over a total of 2-hours [33]. Extant programs also vary considerably in their pedagogical approaches. In the aforementioned meta-analysis conducted by Rudolph and colleagues, only 6 of the studies reviewed (25%) sent home materials with the parents and only 4 (17%) included role-plays or interactive learning strategies [23]. The meta-analysis concludes that parent-focused CSA prevention programs are “generally effective” in facilitating change in parental knowledge, attitudes and behaviors [23]. Yet, parents remain on the fringe of primary CSA prevention efforts, while being a major influence in the lives of their children.

#### Smart parents–safe and healthy kids

In response to some of the documented shortcomings of prior parent-focused efforts, Guastaferro and colleagues [34] developed a behaviorally-based parent-focused CSA prevention program, *Smart Parents—Safe and Healthy Kids* (SPSHK). Informed by social cognitive theory, SPSHK used role play scenarios to emphasize skills related to healthy child sexual development, parent-child communication about sex and sexual abuse, and child safety strategies to protect them from victimization (i.e., vetting the babysitter, monitoring activities inside and outside of the home, as well as online). SPSHK was uniquely designed to be added to existing parent education (PE) programs (e.g., Parents at Teachers, SafeCare, or Incredible Years) as parents who are enrolled in PE, potentially as a result of involvement in the child protective service system, are at increased risk for subsequent child maltreatment, including CSA [22]. Leveraging an existing implementation infrastructure (i.e., PE programs) and targeting at-risk population, was acceptable and feasible [34,35] and effective [36]. However, though a promising advancement in parent-focused CSA prevention efforts, it still left a gap in prevention efforts as there was no evidence-based prevention program for a universal parent audience. Thus, the goal of the current study was to fill this void in the literature by adapting SPSHK for a universal audience using a new program name: *Smarter Parents*. *Safer Kids*.

### Adapting a selective parent-focused CSA prevention program for a universal audience

It would seem that adapting a promising selective prevention approach with empirical support for universal delivery could be an answer to some of the shortcomings of extant universal parent-focused CSA prevention efforts. However, to the best of our knowledge, no parent-focused CSA prevention interventions have transitioned from selective prevention approaches to universal prevention approaches, or vice versa. Additionally, there appear to be no standardized methods or general guidance to inform this adaptation offered in other fields and disciplines—interventions designed as universal or selective have primarily been delivered as such.

In the adaptation process, it was imperative that we retained qualities of SPSHK that set it apart from other programs: delivery in a single session, use of role plays to practice taught skills following principles of social cognitive theory, and provision of materials to the parent that spanned child developmental periods through age 13. Beyond considering what (if any) content needed to be adapted, we weighed implementation constraints of a universally delivered program. Leveraging input from community-partners we decided that to be maximally effective, a universally delivered version of SPSHK must be delivered in a group setting and flexibly delivered online as well as in-person.

### Current study

The purpose of the current study was to identify any necessary modifications to the content of the curriculum and to ascertain whether parents from the general community (rather than referred through a PE program) would have enough foundational knowledge to be successful in the *Smarter Parents*. *Safer Kids*. program. We piloted modifications to the curriculum to foster group rapport and the online delivery system, including online data collection. Using a pre-posttest design, we examined the preliminary efficacy of *Smarter Parents*. *Safer Kids*. in increasing parents’ CSA-related awareness and intention to use protective behaviors. Following the workshop, we conducted brief interviews with parents to learn about the acceptability and feasibility of the curriculum delivered in the group setting, and online. If acceptable and feasible, the eventual addition of an evidence-based universal parent-focused CSA prevention program to child- and community-based CSA prevention efforts holds promise for affecting rates of CSA prevalence. This study reflects the first step in bolstering comprehensive CSA prevention programming.

## Method

### Intervention

SPSHK, the selective prevention curriculum from which *Smarter Parents*. *Safer Kids*. (hereafter *Smarter Parents*) was adapted, is comprised of three segments: (1) healthy child sexual development; (2) parent-child communication; and (3) child safety [34]. Each segment of the handbook is divided into age groups so parents can easily access the most relevant information for their child’s age and developmental range (i.e., 0–2, 2–5, 6–9, 9–12, 13+). In the Healthy Sexual Development segment, parents review typical sexual developmental milestones, behaviors that are atypical and may indicate something abusive or harmful has occurred, and the importance of teaching children anatomical labels for body parts (e.g., vagina or vulva, penis, breasts). In the Parent-Child Communication segment, parents learn why, when, and where to promote open, accurate, and consistent communication about sexual topics. In the Child Safety segment, the parent learns the importance of and how to monitor their child’s activities inside and outside of the home, as well as online. Parents create a safety plan so that their child knows what to do if they are feeling unsafe or if something has happened to them. This segment also reviews the behavioral, emotional, and physical signs of CSA. Parents learn how to react to a disclosure or if they suspect abuse has occurred.

All segments use theory-based role play scenarios to reinforce concepts which are presented in the *Parent Handbook* which each parent receives at the start of the workshop. The facilitator presents a scenario: ‘You drop in unexpectedly to your child’s practice. You see that your child is sitting on the bench looking sad while everyone else is on the field practicing catching. One of the adult coaches is sitting with your child talking to them and the coach has his hand on your child’s leg.’ Which is followed by asking the parents how they would respond. Providers then, using the corresponding scripted *Provider Guidebook*, guide the parent through the correct steps in response to the scenario. For example, related to the sample scenario presented above, parents are guided to recognize and address the child’s discomfort in a one-on-one situation with another adult, practice appropriate intervention, and support the child’s personal boundaries and body safety rules.

#### Adapting for group delivery

Unique to this study was the delivery of *Smarter Parents* in a group setting. Practically, this meant small changes to wording, as well as increased awareness of group and time management. Given the potentially sensitive workshop content combined with the unfamiliar group dynamics, creating rapport and cohesion amongst participants was of significant importance in the adaptation process so as to maximize discussion and engagement in role-playing exercises. To facilitate rapport building, we developed an introductory activity in which participants were invited to share basic information about themselves such as their name, preferred pronouns, age(s) of their child(ren), and one measure they currently take to keep their child safe. This also allowed the Facilitator to record the ages of children for the scenarios selected throughout the workshop. Following the introductions, participants were prompted to reflect on a series of curriculum-specific questions designed to frame the conversation (e.g., “Everyone remembers ‘the talk.’ This was when you were told about sex and sexual development for the first time. Who had ‘the talk’ with you? How did you feel?” and “What are your family’s values about sex and sexual development?”). Reflections were shared in a small discussion prior to initiating the first segment of the curriculum. Any other potential modifications for group delivery were to be identified in the pilot study.

### Participants & setting

**Fig 1:**
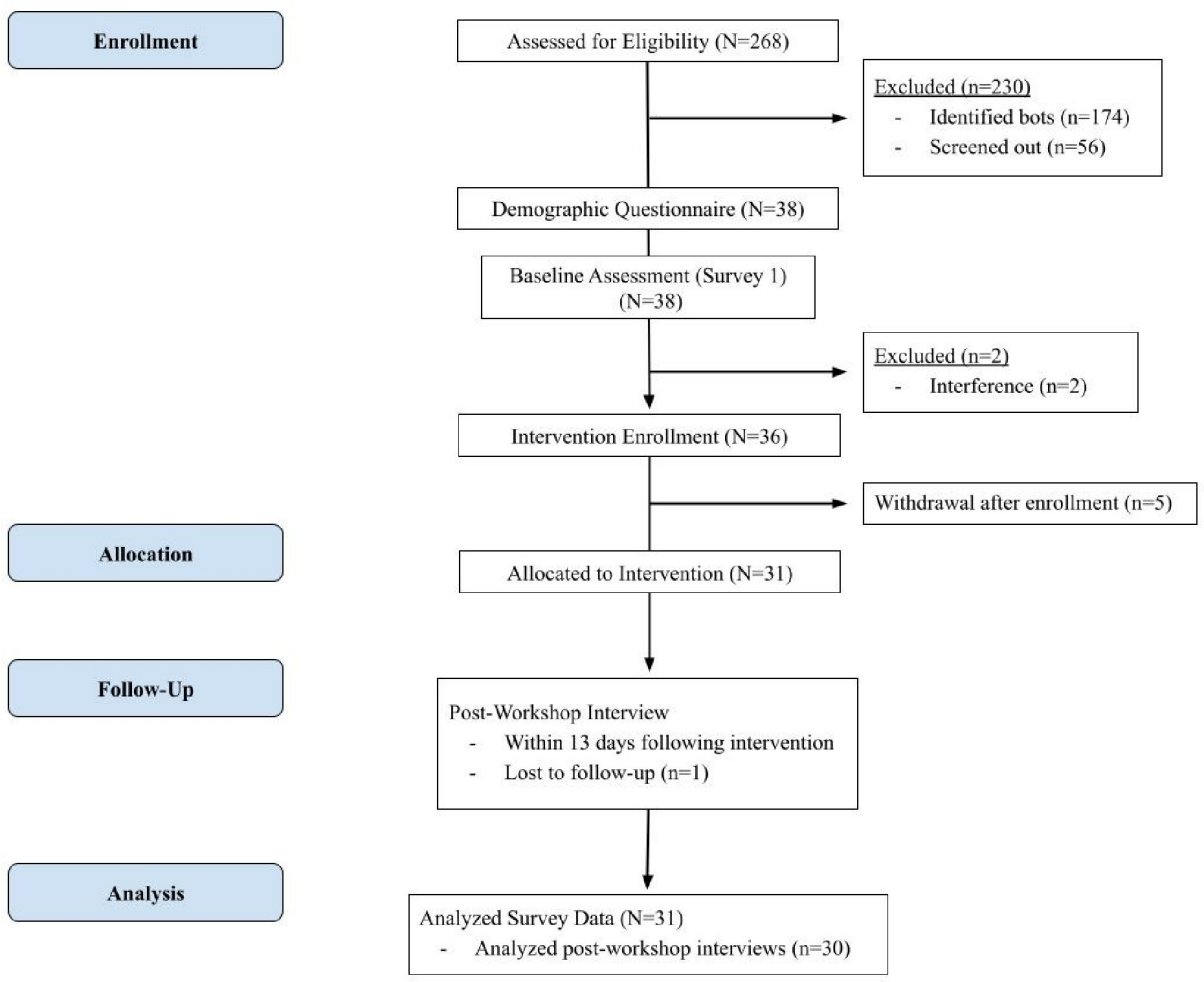
Study flow diagram.

Parents with children under the age of 13 were recruited over a 4-week period between June and July 2023 using online social media platforms managed by the study team and community-based partners. Recruitment posts included a link and QR code to a form through which potential participants could express their interest in being contacted for more information. The ‘Contact Me’ form was the first screening for eligibility: participants had to be older than 18 years old, caregivers of children under the age of 13, and able to read and speak in English. If screened in, eligible candidates were then contacted by a member of our research team via phone. which was useful in identifying robotic agents (bots) that had made it past the initial screening process. During the phone call, participants verbally confirmed their eligibility and were given the opportunity to ask questions about the workshop or study procedures, ultimately providing verbal consent. Verbal consent was permitted as the risks to participants was deemed to be minimal by the University Institutional Review Board. Participants were provided an electronic copy of the consent form with details for the Principal Investigator prior to beginning the first assessment. In total 268 potential participants completed the ‘Contact Me’ form, of which 174 were identified as bots and excluded, and an additional 56 were screened out for other reasons (i.e., not able to contact, phone not in service; Fig 1). Ultimately, 38 participants were successfully recruited and provided consent.

### Experimental procedures

All procedures were approved by the University Institutional Review Board. This mixed-methods pilot study used a pre-posttest design and a brief post-workshop interview. Participants completed online surveys pre- (Survey 1) and post-workshop (Survey 2). After completing Survey 1, participants selected a scheduled workshop date that best suited their availability. Five workshop options were offered to accommodate varying parent schedules and maximize attendance. Approximately 24 hours prior to the workshop all registered participants received via email an electronic PDF version of the *Smarter Parents* Parent Handbook. The Smarter Parents workshops were 120 minutes in duration, conducted via Zoom, and ranged in size from 4 to 9 participants. Each workshop was delivered by two certified facilitators on the research team and was observed by at least three additional members of the research team. Participants were encouraged, but not required, to keep their cameras on to increase engagement and group cohesion. After the Smarter Parents workshop, observers from the research team recorded participation in the workshop which prompted the automatic distribution of Survey 2 to workshop participants. At this time, observers also classified participants into three levels of engagement: low, moderate, or high. To be considered highly engaged in the workshop, participants must have actively participated in practice activities and kept their cameras on throughout the workshop. Participants who were moderately engaged either actively participated in activities or had their camera on, but did neither consistently. Low engagement was defined as not having a camera on or participating in activities. Following completion of Survey 2 participants scheduled a 30-minute semi-structured interview led by a member of the research team. Interviews took place over Zoom and were recorded and transcribed, then reviewed by a team member for de-identification and accuracy.

Participation was incentivized by the sequential distribution of Amazon gift cards sent via email. Each participant received a $50.00 Amazon gift card after completing Survey 1 and a second $50.00 Amazon gift card following the post-workshop interview via Zoom. This phased incentive structure was selected to encourage sustained participation throughout the multiple stages of this study.

### Measures

Participants provided demographic information at Survey 1. The outcome of interest was the Assessment of Smarter Parents (ASK) a 15-item self-report of CSA-related knowledge, attitudes toward CSA prevention (i.e., awareness), and use of protective behaviors (e.g., identify signs of CSA, talking to their child about CSA) [34]. Comprised of two subscales, the ASK has nine awareness-based items (e.g., "Child sexual abuse is a serious problem that only the police should handle. I do not need to be involved") and six behavior-based items (e.g., "My child and I have talked about what to do if someone tries to hurt them"). As it is not possible to determine the actual use of protective behaviors, these items reflect the intention to use behaviors. Responses were measured on a five-point Likert scale, ranging from "Strongly Disagree" (1) to "Strongly Agree" (5) in which higher scores indicate a greater level of awareness and intention to use protective behaviors (note items 1, 6, 9, 10, 12, 14, and 15 are reverse coded). The interview asked parents about their: motivation for enrolling in the program; general thoughts/opinions about CSA prevention; prior experience and knowledge discussing CSA-related topics; and overall experience in the *Smart Parents* session and the content provided (e.g., “What was most helpful to learn in the Smart Parents session?”). Parents were also encouraged to provide suggestions for how the workshop could be effectively advertised in the future.

### Analytic approach

All data, including the screening information, were collected via REDCap [37] and statistical analyses were used conducted using R software version 4.3.1. As the goal of the study was acceptability and feasibility of the universal adaptation delivered in a group setting, the main focus was qualitative data from the post-workshop interview. Beyond piloting the procedures for online quantitative collection, we compared pre-posttest means on the ASK to examine potential efficacy. The pilot study was not designed to test hypotheses, but rather to generate hypotheses for a future trial [38]. As such, only descriptive statistics were computed to summarize the data. Participants were allowed to skip any questions they did not wish to answer resulting in varying *n*’s per item.

Interviews were analyzed using qualitative content analysis methods [39]. Themes and corresponding codes emphasized the purpose of the study: to identify necessary modifications to the universal curriculum (content or presentation) and to assess acceptability and feasibility. Three themes, and subsequent subcodes, were identified: (1) parent as agent of prevention, (subcodes: prior action, prior knowledge, new knowledge, confidence, action); (2) curriculum (subcodes: content, design, changes); and (3) engagement (subcodes: marketing/promotion, parent motivation, future use). To ensure the reliability of the analysis, six independent coders initially coded three transcripts independently and then met to evaluate inter-coder reliability. Adequate reliability was defined as greater than or equal to 80% agreement in extracts pulled from each code. To achieve reliability coders met to resolve any disagreements and updated the codebook accordingly. The remaining transcripts were then divided among coders, such that each transcript was coded by two members of the research team. Codes and reliability were reviewed by the lead author throughout.

Though 38 participants were successfully recruited and consented, 2 were excluded prior to the workshop delivery as a result of interference (i.e., registering for the workshop under multiple names). After signing up for the workshop (*N* = 36), 5 participants withdrew for various reasons (i.e., no longer interested, lack of time, schedule conflict) leaving a final analytic sample of 31 participants who completed Survey 1, the Smarter Parents workshop, and Survey 2 (i.e., allocated to intervention). One participant declined to participate in the debrief interview resulting in an analytic sample of *n* = 30 transcripts for qualitative analyses.

## Results

Demographic characteristics of the sample are displayed in Table 1. The majority of participants were female (55%), White (53%), with a mean age of 36.5 years (SD = 5.8; Range: 29–50), having attained a college or advanced degree (93%), and the majority (84%) reported being married or living with a partner. Income varied; of the 16% of participants reporting an annual household income <$40,000, one reported between $5,000 –$9,999, one between $10,000 - $14,999, two between $15,000 - $24,999, and one between $25,000 - $39,999. The average number of children per participant was 1.9 (SD = 0.97; Range = 1–5) with ages ranging from newborn to +13. Slightly over a quarter (26%) of the sample reported receiving at least one form of financial assistance, the most commonly reported of which was Medicaid (n = 6). Of the 30 parents who completed an interview, the majority reported finding the study ad on social media, specifically Facebook (*n* = 16), LinkedIn (*n* = 7), and Reddit (*n* = 1). Others mentioned personal connections to study team (*n* = 5) or with a partnering community-based organization (*n* = 1).

**Table 1:** Sample characteristics of participants of standalone pilot study 2023 (*N* = 31).

	*N*	*%*
Gender		
Male	14	45
Female	17	55
Marital Status		
Married or Living w/ Partner	26	84
Single	5	16
Hispanic/Latino	3	10
Race		
White	16	53
Black	14	47
Highest Education Attained		
Some College	2	6
College Graduate	14	45
Advanced Degree	15	48
Household Income		
$5,000 - $39,999	5	16
$40,000 - $59,999	5	16
$60,000 –$74,999	6	19
$75,000 - $100,000	4	13
≥ $100,000	11	36
Receiving Financial Aid	8	26
# of moves in the past year		
0	15	48
1	10	32
2–3	5	16
Age of All Child(ren), years		
0–1	5	9
2–5	11	19
6–8	15	25
9–12	19	32
>13	9	15

### Preliminary efficacy

Item-level changes on ASK items from pre- to post-workshop are presented in Table 2. Overall, participants’ mean score on the CSA-related awareness subscale nominally increased from 36.9 to 39.2 (Δ+2.3) and the mean score for intention to use protective behaviors nominally increased from 20.2 to 23.6 (Δ+3.4). Out of all 15 items, the greatest mean score change (Δ+1.0) was observed for Item 3 (e.g., “I know what signs to look for that suggest my child may have been sexually abused”). In contrast, the item with the lowest mean score change (Δ+0.03) was Item 1 (e.g., “Child sexual abuse is a serious problem that only the police should handle, I do not need to be involved”). Item 4 (e.g., “Most sexual abuse victims are abused by someone they know.”) decreased by 0.03 points from pre to post workshop; however, the means were above 4 indicating a high level of agreement overall.

**Table 2:** Parents’ CSA-related awareness and intention to use protective behaviors as measured by the assessment of SmartParents knowledge (*N* = 31).

	Pre-Workshop Mean (SD)	Post-Workshop Mean (SD)	Δ Survey 1 to Survey 2	Effect Size (Cohen’s *d*)
***Awareness***				
1. Child sexual abuse is a serious problem that only the police should handle. I do not need to be involved.	4.26 (1.21)	4.29 (1.42)	+0.03	+0.02
2. Children should be taught the correct names for their private parts (e.g., penis, vagina).	4.68 (0.54)	4.97 (0.18)	+0.29	+0.72
4. Most sexual abuse victims are abused by someone they know.	4.35 (0.61)	4.32 (0.98)	-0.03	-0.04
6. The only time a parent should talk to their child about sex is when he/she reaches puberty.	3.77 (1.33)	4.35 (1.05)	+0.58	+0.48
8. It is okay if my child does not want to hug an adult, such as a family member.	4.39 (0.76)	4.68 (0.48)	+0.29	+0.46
10. My children might become sexually active because I talk to them about sex.	3.87 (1.09)	4.06 (1.18)	+0.19	+0.17
11. I know what healthy sexual development is.	3.87 (0.67)	4.52 (0.57)	+0.65	+1.04
13. It is okay to ask for a background check for a new babysitter.	4.45 (0.77)	4.71 (0.78)	+0.26	+0.34
15. Children should learn about how to prevent sexual abuse only in schools.	3.32 (1.58)	3.61 (1.67)	+0.29	+0.18
***Behaviors***				
3. I know what signs to look for that suggest my child may have been sexually abused.	3.52 (1.09)	4.52 (0.57)	+1.00	+1.15
5. I have talked to my child about how to protect themselves from being sexually abused.	3.48 (1.15)	4.06 (1.00)	+0.58	+0.54
7. My child and I have talked about what to do if someone tries to hurt them.	3.61 (1.12)	3.81 (1.11)	+0.20	+0.18
9. I do not know what signs to look for that suggest my child may have been sexually abused.	3.06 (1.18)	4.10 (1.21)	+1.04	+0.87
12. I have not talked to my child about sexual abuse.	3.19 (1.38)	3.58 (1.34)	+0.39	+0.29
14. My child and I have not talked about what to do if someone tries to hurt them.	3.35 (1.33)	3.58 (1.46)	+0.23	+0.16

Participants who were categorized as low-engagement (*n* = 7) increased their mean score of awareness by 5.1 (31.9 to 37.0) and in intention to use protective behaviors by 3.2 (20.9 to 24.1) from pre- to post-workshop. In comparison, participants categorized as moderately-engaged (*n* = 9), increased their mean score of awareness by 1.0 (38.3 to 39.3) and intention to use protective behaviors by 2.4 (21.3 to 23.7). Participants categorized as high-engagement (*n* = 15) increased their mean score of awareness by 2.3 (38.5 to 40.8) and intention to use protective behaviors increased by 3.8 (19.3 to 23.1) from pre- to post-workshop.

### Parents’ qualitative input

Overall, parents shared overwhelmingly positive reactions to the workshop:

I really enjoyed some of the very tangible ways to communicate the age-related suggestions too, of like how to build upon and create those building blocks for these conversations. Like even—even with my 16-month-old, which was surprising to me, too, to think about all the ways that I could start doing that now, when it doesn’t feel as relevant… or it didn’t. But now it does feel relevant, for building this conversation. (Participant 441)

#### Parents as agent of prevention

Overall, interviews suggest participants initially felt inexperienced and uncertain how they had addressed CSA with their children prior to the workshop (Table 3). In part, because of a lack of discussion in their childhood home: “*It was more of a taboo topic*. *I honestly don’t remember having these discussions at home*” (Participant 45). However, parents reported gaining valuable knowledge from the workshop, which led to a significant shift in their perspective as parents. One parent shared: “*Maybe what I had before was maybe like I was aware of maybe like child sexual violence*, *but I could not maybe*, *like*, *know how to maybe respond to such a situation*” (Participant 46). Parents highlighted the importance of learning to identify age-specific red flags and warning signs while closely observing changes in their children’s behavior. One parent shared: “*It’s just more than using my eyes to watch them*. *You need to tell them about it*. *You need to educate them*. *And I think I learned a lot*” (Participant 88).

**Table 3:** Exemplar quotes for the theme of parent as agent of prevention (*n* = 30).

Subcode	Positive/Majority	Dissenting/Alternative
Prior Action	As I said, I take it upon myself to be very close to my children. So, they could be very comfortable confiding in me. So, it’s something I know already that I need to be very close to my here. (Participant 72)	I think I’ve had difficult moments talking to my kid about sex. And I think this is influenced by how I was raised when I was raised. (Participant 75)
Prior Knowledge	I think that as parents sometimes we feel guilty, sometimes we blame ourselves for some situations at home, but no one gave us a handbook. So, when you make an effort and the fact that it’s been recognized, and someone is telling you that you’re trying, and you could do better. I think it’s very encouraging, because you don’t get paid for being a parent, and it’s one job that is crazy. (Participant 33)	I’m in a similar field as, so I wasn’t surprised by the statistics. It didn’t really change my perception of risk. (Participant 462)
New Knowledge	It was a taboo and undiscussed topic, absolutely. Yes, it was something that my parents and my family, you know, pretty much relied on the school system to provide that education for us. (Participant 54)	Yeah, definitely, it’s always there…It was just a family discussion. (Participant 26)
Confidence	I get to know that children are supposed to be well informed about those sensitive topics. And it’s quite pertinent that they get to learn all this topic from home. Initially, I actually thought that it wasn’t my responsibility to inform them about it. (Participant 32)	I have worked in child welfare for 20 years. So, I don’t know if I learned anything new on that end, but I did learn how to frame things without putting more anxiety on my daughter. (Participant 96)
Action	I’m actually right now, a scale of 1 to 10, I’m actually like 8 compared to 4, so it did a lot of good for me. (Participant 36)	I would say, somewhat confident. I don’t think I could ever say fully confident, because you don’t really know how they’re gonna respond. But I do feel comfortable enough talking to my kids. (Participant 58)

#### Curriculum

The majority of parents found the curriculum, including the Parent Handbook, beneficial and easy to follow (Table 4): “*I think that it was very easy to understand*, *very self-explanatory*…… *I’ve made mistakes and I think*, *having that handbook just made it so easy for me to understand*, *and I didn’t feel uncomfortable in any kind of way*” (Participant 33).

**Table 4:** Exemplar quotes for the theme of curriculum (*n* = 30).

Subcode	Positive/Majority	Dissenting/Alternative
Content	To me the most interesting part was the breakdown of ages, like what signs to look out for. Because, you know, I figure like, oh, it’s going to be the same signs. And, obviously I came across a new, different things that I would never think to look out for. (Participant 94) The take away boxes that we talked about, and the scenarios were perfect because it gave me a chance to practice. I think the scenarios were amazing. I think that was a great way to do the learning. (Participant 35)	I feel like there was so much information, and we did go through different scenarios, but I wonder if there’s a way to break it up a little bit. Hopefully this is helpful, like, I don’t have anything to—like, something I can pinpoint. I thought maybe if it was a PowerPoint instead of the workbook, like we could look at a PowerPoint together and kind of talk about it. (Participant 281)
Design	It’s like a parent guideline on how to be able to say some certain things right, and you always going to have it to refer back to. (Participant 33)	I feel like, if you’re maybe an older parent, it might be a little bit challenging, because, you know, they’re not as tech savvy. But for me it was very easy to follow along. (Participant 94)
Changes/Modifications	I feel like the two hour was a little tough, being present, that entire time, even if there was a break or something. ‘here’s a 5-minute break’ (Participant 45)	I actually wish that we could have gone for more, more time. (Participant 351)

Related to the group format, numerous participants highlighted the gainful experience of group interaction and the ability to hear answers from parents with children of different ages than their own. For example, one participant noted “*I thought that was where the conversation with the group was really helpful*… *some more experienced parents talk through what’s worked for them*, *I actually really liked that…*” (Participant 441). Another participant: "*But being a in a midst of other people*, *you know and getting to learn from their experience and getting to learn from their own world and all of these other things*, *that’s the favorite of it*" (Participant 72). Many participants found the childhood sexual development and parent-child communication sections as particularly memorable with tangible examples to implement the learned skills. Many participants indicated a preference for virtual sessions with the Parent Handbook as an electronic resource, and did not feel challenged by accessing zoom and the handbook simultaneously. Lastly, participants similarly suggested a workshop less than 120-minutes in duration, although most did not find it a barrier to their attendance.

Though the pilot was conducted virtually for practical reasons, we asked parents if they preferred in-person, virtual, or would not mind either delivery modality. The vast majority (*n* = 24) indicated they would prefer the virtual option, citing ‘accessibility’ as the driving feature: “*It’s kind of*, *with having a toddler*, *it’s kind of hard for me to go do in-person things*” (Participant 86) and “*I like the virtual*. *It’s just easier than going somewhere*, *you get more people involved*, *at the same time I think it’s more convenient trying to do it for scheduling purposes*” (Participant 45). The one participant who indicated a preference for in-person delivery explained: “*you can to get to learn more from different people*” (Participant 351). Five participants indicated ambivalence with delivery modality:

I always like in person, but that’s just me. I don’t know if that would be possible again, because I think we did have a couple of people from all over. I always prefer in person over the so rather than over the phone or zoom. So that would be a preference of mine…. it’s still important to have online, because again, everyone can access it, and they can do it more at a convenient like a schedule that works for them. So, that’s the world we live in now. I think it is important to have it available online. (Participant 58)

#### Engagement

Parents reported that their motivation for participation was derived from a desire to educate themselves on how to best protect their children through both program content (e.g., “*To educate myself*, *educate my children*, *how to be safe*” (Participant 45)) and dialogue with other parents (e.g., “*You need to also learn from people*, *you need to get people’s insights so you could also be able to provide the best fatherhood*” (Participant 88)). Several parents noted that they intended to share the handbook as a “tool” with other parents and spoke about referring back to it as their children age. One participant shared: “*It’s like a parent guideline on how to be able to say some certain things right*, *and you always going to have it to refer back to*. *So*, *I think*, *having that whole process was something that I wish I had earlier*” (Participant 33). Another participant shared: "*Sometimes*, *even in my community there are some things we neglect*. *There are some things we overlook and being in this*, *in the forum we were*, *I felt like now I can be the champion in my community*. *So*, *that for me is a win maybe for my community*." (Participant 75).

When discussing suggestions for future promotional strategies, parents encouraged increasing program awareness through social media and word-of-mouth among fellow parents: “*I know social media is great*, *sometimes*, *to get information out there*. *Word of mouth*… *list 3 to 5 names*, *other moms who might feel comfortable that I could reach out to*” (Participant 281). Other parents mentioned the skills strengthened through this workshop could be beneficial for recruitment efforts: “*I think just highlighting that this really works to give you communication skills*, *and create that openness so your child feels like they can talk to you*. *I think that is a very compelling angle for me as a parent*” (Participant 441).

## Discussion

Parents have long been underutilized in the prevention of CSA. This study sought to adapt a promising selective prevention approach for universal delivery. As there are no guides for this type of adaptation in the literature, the process described herein may serve as an initial blueprint for other research teams wishing to adapt a selective or targeted intervention for a universal audience of parents, or caregivers.

Retaining the qualities of the original version, the adapted universal curriculum *Smarter Parents*. *Safer Kids*. was delivered in a single session, used behavioral skills training via role play scenarios, and was developmentally comprehensive. The distinguishing feature of *Smarter Parents*. *Safer Kids*. was the group delivery format. Qualitative findings indicate that parents found the group format to be an asset to learning and they felt as though they benefited from participating. These subjective findings are supported by quantitative increases in awareness and intention to use protective behaviors, though the statistical significance of these increases remains unknown. Overall, there was a high degree of acceptability for the universal curriculum. The findings of this study add to a body of growing evidence by the team [40,41] that indicates resource intensive, large scale adaptations of CSA prevention programs may not always warranted.

As a pilot study, the feasibility of studying the curriculum in a larger evaluation trial was the central focus. Preliminary efficacy estimates suggest that participation moves outcomes in the desired direction, and further evaluation in a rigorous trial is warranted. Due to the study design and sample size, the results presented herein should not be interpreted as conclusive [38]. Related to future studies, a number of procedural challenges remain unsolved. In particular, bots posed a significant challenge throughout the initial recruitment period, with 174 out of 268 entries in the ‘Contact Me’ form identified as bots. This is a plight of other studies using online participant recruitment. Social media, in particular, often attracts bots seeking financial incentive [42]. To combat the bots in the current study, the team flagged data on the contact form that shared variations of the same phone number or email address. Entries submitted within minutes of each other were also flagged for review. After the first round of bots, the team added ‘human questions’ to the contact form (e.g., “What color is a lemon?”), but some bots were able to bypass this question by chance. We also enacted reCAPTCHA measures as a means of preventing bots as recommended in the literature [42]); however, if using the same IP address, the reCAPTCHA was bypassed the second time the link was tried. Related, as evidenced by high baseline ASK scores, the sample who participated in this pilot study were moderately knowledgeable and motivated to participate. Engaging parents who are less knowledgeable or motivated, and thus harder to reach, is a priority for future research. Across the board, research must overcome the challenge of online recruitment, but must also consider what style or wording on study ads is best to foster participation.

As a pilot study, the acceptability and feasibility findings are encouraging though there are a few limitations that should be discussed. The sample had a degree of variability among participants in relation to parent demographic characteristics, but the recruitment challenges (i.e., bots) created more of a convenience sample than initially planned. The findings may not be representative of all parents who may participate, but the findings are also not designed to be generalized in a conclusive manner.

The first step in bolstering comprehensive CSA prevention programming, the novel universal parent-focused curriculum described herein will add to an existing menu of well-established prevention programs as the evidence for this specific curriculum evolves. A community that implements universal parent-focused prevention, in addition to programs for at-risk parents [36], and school-based child-focused programs [41,43,44] in a coordinated and systematic manner has the greatest potential for seeing reductions in the rates of CSA. These programs, however, cannot be implemented sporadically if impact is desired–it will require an investment of considerable community resources and changes to policy. However, prevention is possible and even more likely when a comprehensive approach to prevention–that is one that includes children, parents, and the community—is adopted.
